# Applying Next-Generation Sequencing Platforms for Pharmacogenomic Testing in Clinical Practice

**DOI:** 10.3389/fphar.2021.693453

**Published:** 2021-08-25

**Authors:** Alireza Tafazoli, Henk-Jan Guchelaar, Wojciech Miltyk, Adam J. Kretowski, Jesse J. Swen

**Affiliations:** ^1^ Department of Analysis and Bioanalysis of Medicines, Faculty of Pharmacy with the Division of Laboratory Medicine, Medical University of Bialystok, Bialystok, Poland; ^2^ Clinical Research Centre, Medical University of Bialystok, Bialystok, Poland; ^3^ Department of Clinical Pharmacy and Toxicology, Leiden University Medical Center, Leiden, Netherlands; ^4^ Leiden Network of Personalized Therapeutics, Leiden, Netherlands; ^5^ Department of Endocrinology, Diabetology and Internal Medicine, Medical University of Bialystok, Bialystok, Poland

**Keywords:** pharmacogenomics, clinical implementation, next generation sequencing, clinical practice, PGx testing

## Abstract

Pharmacogenomics (PGx) studies the use of genetic data to optimize drug therapy. Numerous clinical centers have commenced implementing pharmacogenetic tests in clinical routines. Next-generation sequencing (NGS) technologies are emerging as a more comprehensive and time- and cost-effective approach in PGx. This review presents the main considerations for applying NGS in guiding drug treatment in clinical practice. It discusses both the advantages and the challenges of implementing NGS-based tests in PGx. Moreover, the limitations of each NGS platform are revealed, and the solutions for setting up and management of these technologies in clinical practice are addressed.

## Introduction

### The Importance of Pharmacogenomics in Modern Medicine

Pharmacogenomics (PGx) utilizes individuals’ genomic profiles to identify those who are at greater risk for adverse drug reactions or ineffectiveness. Many studies clearly indicate that drug-related genes, also referred to as “pharmacogenes,” in the human genome contain extensive functional genetic variations (FGVs) and that different alleles are associated with diverse outcomes of drug treatments (Madian et al., 2012; Guchelaar, 2018; Suarez-Kurtz and Parra, 2018). Around 97–98% of people have at least one actionable FGV in their drug-related genes. In addition, the possibility of the presence of a genetic variant which could result in a loss of function (LOF) variant in pharmacogenes is 93% for every individual (Schärfe et al., 2017). Hence, the identification of the different genetic variants associated with the drug metabolism would impact on the prescription of medication, allowing for the selection of the right drug and dose, thereby reducing the potential adverse effects or the therapeutic inefficacy. For clinical interpretation of PGx tests, the Clinical Pharmacogenetics Implementation Consortium (CPIC) and the Dutch Pharmacogenetics Working Group (DPWG) guidelines are available as well as FDA drug-gene interaction recommendations. CPIC originally started as a shared project between PharmGKB and the Pharmacogenomics Research Network (PGRN) in 2009, and DPWG was launched in 2005 by the Royal Dutch Pharmacists Association. The two consortia have developed and published recommendations for numerous gene-drug interactions (JJ Swen et al., 2011; Relling and Klein, 2011). Both CPIC and DPWG provide updated, evidence-based, free access guidelines to facilitate and accelerate the establishment of a link between the results of PGx tests and specific dose recommendations. Nowadays, an increasing number of specified PGx tests are available in specialized CAP/CLIA approved clinical pharmacology/genome analysis centers around the world and can be found in the genetic testing registry (GTR, https://www.ncbi.nlm.nih.gov/gtr/) (Jiang and You, 2015).

**FIGURE 1:**
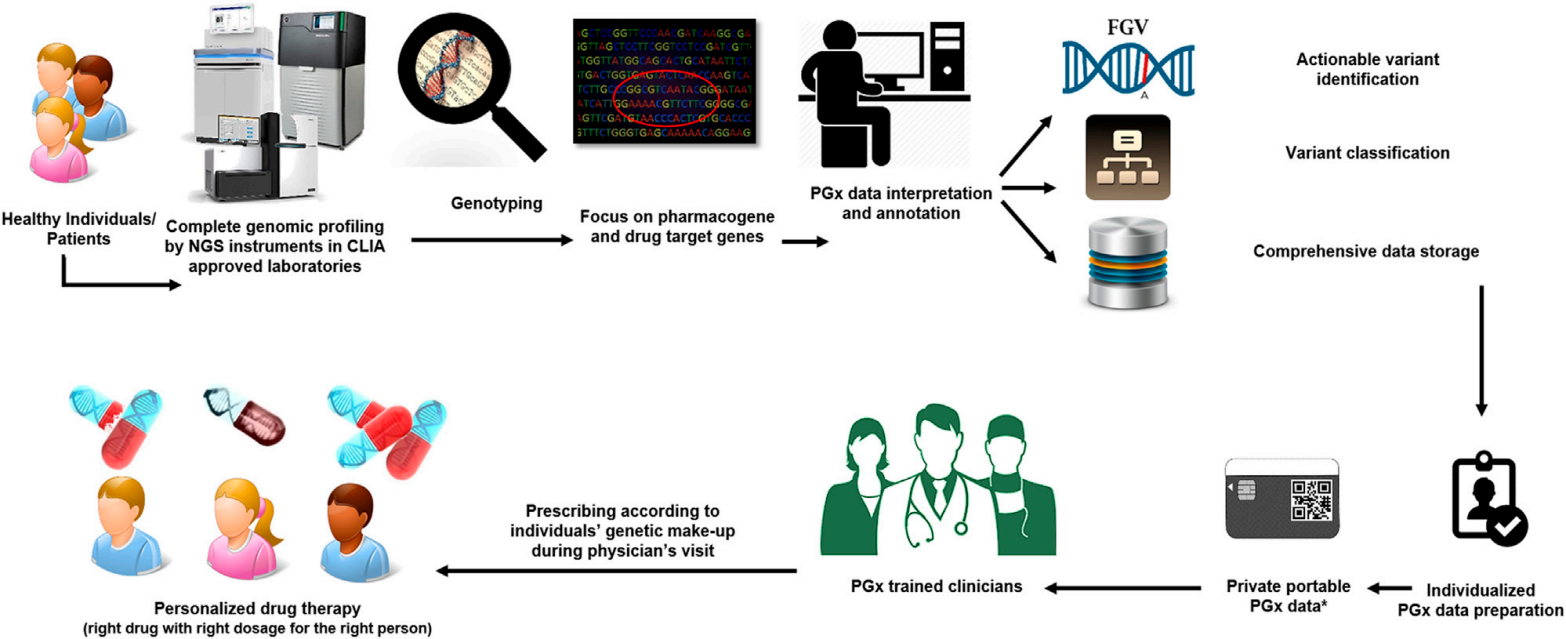
A prospective view of the use of pharmacogenomics in modern medicine. Every person (sick or healthy individuals) will undergo comprehensive genomic screening before going to the physician/clinicians. The genetic variations in all pharmacogenes of an individual will be identified through data annotation and visualization by specific bioinformatics tools. The final report for each individual will be available through a private portable PGx electronic card. PGx trained clinicians will use an individual’s genetic make-up report to tailor treatment to the patient’s needs.

The introduction of next-generation genome sequencing in PGx practice is an interesting and promising, albeit challenging, step. Currently, the field of PGx is moving from reactive testing of a single gene towards scanning an entire panel of genes involved in drug absorption, distribution, metabolism, and excretion (ADME) before prescribing (pre-emptive genotyping) by applying different types of next-generation sequencing (NGS) platforms (Bielinski et al., 2014; van Der Wouden et al., 2019). The results include all the PGx-related genetic variants in the genome which will be utilized to prepare drug dosing recommendations based on the predicted phenotype provided by the sequencing tests (Figure 1).

While the topic is highly popular and an overview of the current state of the NGS technologies for use in PGx testing has been offered in the literature previously (Schwarz et al., 2019), this article will discuss the challenges of detecting specific types of variants in PGx and interpreting such data in clinical practice. Solutions for the establishment and management of NGS devices in clinical practice are also addressed. A number of useful tables that provide detailed NGS-PGx-related information are also included. To aid with the terminology used throughout this manuscript, we included a concise glossary of NGS-related terminology in Appendix 1.

## How Can We Use NGS for PGx Analysis?

In this section, we firstly discuss the SNP-based PGx testing, which is currently the most frequently used test in the clinical PGx profiling of individuals, followed by targeted sequencing and whole-exome and whole-genome sequencing (WES/WGS).

### SNP-Based PGx Testing in Clinical Practice

Fast, accurate, and inexpensive genotyping methods are key to the implementation of PGx in clinical practice. Currently, specific genotyping methods which mostly utilize different types of SNP-based genotyping approaches including real-time PCR with TaqMan probes and restriction fragment length polymorphism (RFLP) technique as well as gene panel-based genotyping methods such as ADME arrays are used in everyday clinical practice (Dorado et al., 2005; Johnson et al., 2012; Larsen and Rasmussen, 2017; Rasmussen-Torvik et al., 2017; Lemieux Perreault et al., 2018; Hippman and Nislow, 2019). In principle, genome-wide genotyping arrays such as Infinium Global Screening Array (GSA) could be used for routine PGx testing but are not yet commonly applied for this purpose. While the technology is still developing, the main limitation is that the identification of the structural PGx variants such as Copy Number Variations (CNVs) and hybrid genes as well as CYP2D6/7 is mostly ignored. Moreover, the variants in the pharmacogenes that are tested are limited to currently known and common alleles. Although several versions of arrays are being enriched with more specific PGx variants (thousands of drug-related biomarkers) (Arbitrio et al., 2016; Thermofisher.com/Pharmacoscan, 2018; Illumina, 2020), no phasing information will be obtained through these tests, which makes it more challenging to provide an accurate phenotype prediction.

Hence, the properties of NGS technologies make them an interesting approach to performing clinical PGx testing. In recent years, several investigators have explored different approaches utilizing NGS platforms, namely, targeted sequencing, WES, and WGS in pharmacogenomics. Table 1 shows several studies stratified by different approaches.

**TABLE 1:** Summary of the recent studies that used the NGS technologies for functional PGx variant detection.

Study objective	n =	Applied NGS platform(s)	Covered drug-related genes	Identified variants	Result	Reference
Platform validation and variant discovery	3 × 96	Targeted sequencing	84	SNVs	A custom-designed panel (PGRNseq) could be an ideal platform for both the common and the rare PGx variants identification in large cohorts and suitable for the clinical tests	Gordon et al., 2016
Platform validation and variant discovery	376	Targeted sequencing	114	SNVs	Targeted sequencing panels are ready-to-use platforms for comprehensive pharmacogene profiling including common plus rare variants in ADME core genes towards the implementation of the personalized medicine	Han et al., 2017
Platform validation	2 (cell culture)	Targeted sequencing	3	SNVs	Variants and haplotype detection of challenging ADME genes were successfully achieved	Ammar et al., 2015
				CNVs		
				InDels		
Platform validation and variant discovery	235	Targeted sequencing	100	SNVs	Designed PGxSeq panel with high accuracy identified clinically relevant variants in 39 genes including CYP2D6 CNV and UGT1A1*28 TAA repeats in the promoter. The allele frequency and the homozygosity were also determined	Gulilat et al., 2019
				CNVs		
Platform validation and variant discovery	150	Targeted sequencing	340	SNVs	Panel-based NGS pipeline developed and revealed 7,273 novel variants in 340 ADME genes of 150 Caucasian liver donors with an accuracy of >99%. The functional prediction allowed for the prioritization of the variants for further analysis	Klein et al., 2019
				Small InDels		
Validation of known variants	60	Targeted sequencing	20	SNVs	Prediction model of the atorvastatin plasmatic concentrations in healthy volunteers through the sequencing results explained well	Cruz-Correa et al., 2017
				InDels		
Platform validation	98	Targeted sequencing and WGS data	19	SNVs	The concordance between the two platforms estimated to >97% for identified variants. The CNVs concordance in CYP2D6 gene also demonstrated 90% of accuracy. 95 children had at least one clinically actionable pharmacogenetic variant	Cohn et al., 2017
				CNVs		
Validation of known variants	1,583	Whole-exome sequencing data	11	SNVs	At least one actionable phenotype was present in 86% of individuals. Repurposing WES data can yield meaningful pharmacogenetic profiles for 7 of 11 important pharmacogenes, which can be used to guide the drug treatment	van der Lee et al., 2020a
Validation of known variants	94	Whole-exome sequencing	3	SNVs	Diagnostic genotyping identified PGx variants in CYP2C19, CYP2C9, and VKORC1 genes in 91% of all cases. Of this, 20% indicated potential immediate effects on the currently used medications	Cousin et al., 2017
Platform validation	36 + 12	Whole-exome sequencing	36	SNVs	High concordance revealed through cross-comparison of WES and other platforms as well as the MiSeq amplicon sequencing data and the IPLEX ADME PGx panel. WES was introduced as a promising tool in PGx profiling with a low error rate of <1%	Wee Chua et al., 2016
				InDels		
Platform and discovery rate validation	2504 of WGS data + 59,898 of WES data	WGS and WES data	208	SNVs	The population-specific deletion and the duplications were revealed in 97% of the analyzed subjects and the related frequencies were reported and confirmed via Sanger sequencing	Santos et al., 2018
				CNVs		
Platform validation and variant discovery	1,000 Genomes data	Whole-genome sequencing data	160	SNVs	Putatively functional variants within known pharmacogenomics loci identified that could account for association signals and represent the missing causative variants underlying drug response phenotypes	Choi et al. (2019)
				InDels		
Variants validation and discovery	547 individuals from in-house cohort data + gnomAD data	Whole-genome sequencing data	11	SNVs	For improved precision medicine, PGx testing should move towards WGS-based approaches as a feasible and most comprehensive method	Caspar et al. (2020)
				InDels		
Platform validation	44,000 biobank participants	WGS and WES data + microarray data	11	SNVs	WGS and microarray demonstrate more concordances for the obtained results. WES is not suitable for PGx preemptive predictions. However, the microarrays are more cost-effective than the sequencing platforms. Overall, the implementation of the PGx tests and the recommendations may affect at least 50 daily drug doses per 1,000 inhabitants	Reisberg et al. (2019)
				CNVs		
Variant discovery	3	Targeted sequencing	16	SNVs	The functional alterations and variants with potential impact on anti-TNF drug response successfully introduced by rapid, sensitive, and cost-effective NGS-based pharmacogenetics methodology	Walczak et al., 2019
Variant discovery	392	Whole-exome sequencing	21,000	SNVs	Exome sequencing revealed novel genetic loci with a strong association with on-treatment reactivity and hereditability of platelet and clopidogrel response	Price et al., 2012
				InDels		
Variant discovery	482 + 7	Whole-genome sequencing	231	SNVs	17,733 ADME variants/individuals detected. In addition to known PGx markers, 1,012 novel variants with potential deleterious function identified in exons, introns, gene promoters, and proximal regulatory regions	Mizzi et al., 2014
				InDels		
				Tandem substitutions		
Variant discovery	100	Whole-genome sequencing	437	SNVs	The analysis revealed 227 common and 466 rare population-specific potentially functional SNVs	Sivadas et al., 2017

### Targeted Sequencing Panels

Research into PGx over the years has resulted in the identification of numerous genes which may play an essential role in drug metabolism, transport, and targeting in the human body. However, not all of them are strongly associated with drug response phenotypes and therefore CPIC and DPWG only provide clinical recommendations for specific variants in well-known pharmacogenes.

Gordon et al. developed the PGRNSeq panel as a balance between cost, throughput, and depth of coverage. The panel included clinically actionable CPIC genes as well as genes for which little was known, although a primary association with the PGx trait existed. It was concluded that the PGRNSeq panel is suitable for both the clinical investigations and the discovery studies. However, some non-coding parts and complex structural variants for specific pharmacogenes (including CYP2A6, CYP2D6, and HLA-B) alongside better computational resources for data interpretation remain to be developed. In a similar approach, Han et al. developed an unbiased and broad-range NGS panel and suggested that the utilization of such panels may be a valuable tool in the comprehensive study of PGx genes. The selection of genes for inclusion in the panel was based on the pharmaadme and Www.pharmaadme.org database (Gordon et al., 2016; Han et al., 2017).

Customized PGx panels can also serve as a highly accurate approach to variant detection in the clinical PGx testing. Gulilat et al. developed a targeted exome panel, named PGxSeq, for capturing both SNVs and CNVs in pharmacogenes. They demonstrated that PGxSeq could be employed as a reliable tool for common and novel SNVs alongside CNV detection in pharmacogenes in clinical use. However, a limitation of the work was that the validation was restricted to 39 loci in 16 genes in specific population samples. Moreover, pharmacogenetic variants in non-coding and regulatory parts were not included (Gulilat et al., 2019). A comprehensive PGx panel that includes all coding regions, adjacent introns, and 5′ and 3′ UTRs in flanking sequences of 340 ADME genes has recently been developed by investigators in Germany. The identification of genes for inclusion in the panel was based on multiple sources including PharmaADME, PharmGKB, and ADME-related genes from the literature. Compared with other genotyping methods, accuracy was high, with >99% correct calls. The obtained data allowed for the covering of coding and functional non-coding parts and provided related data for both common and rare variants in addition to revealing novel associations. The detection of some limited InDels and integration of rare variants into PGx by the current computational predictors alongside the sample size were reported as limitations of the panel (Klein et al., 2019).

### Long-Read Sequencing for Gene Panels

Several PGx genes involve complex variants such as tandem repeats, pseudogenes, and CNVs. Long-read sequencing approaches (on average over 10 kb in one single read) have been used previously in the profiling of different complex genomic loci and have been proposed for the identification of such challenging genomic areas in PGx (Ardui et al., 2017; Mantere et al., 2019; van der Lee et al., 2020a). In this field, Ammar et al. applied long-read sequencers to identify PGx variants and haplotypes in three challenging pharmacogenes: CYP2D6, HLA-A, and HLA-B. The constructed haplotypes were confirmed by HapMap data and statistically phased Complete Genomics (WGS data from the public 69 genomes project) and Sequenom genotypes (for 36 SNP, InDels, and CNVs for CYP2D6). The results demonstrated the potential of long-read sequencing in clinical PGx (Ammar et al., 2015). In addition to haplotyping, variant phasing is also a challenge in PGx. Long-read sequencing has also been employed to resolve phasing issues and provide a solution to the accurate genotyping of complex PGx genes. Yusmiati Liau et al. utilized the GridION platform for sequencing and haplotyping of the entire CYP2D6 gene. Known and new alleles and subvariants plus duplicated alleles were assigned accurately with correct phasing. The approach also demonstrated the capability of processing multiple samples simultaneously and appeared to be a time- and cost-effective method (Liau et al., 2019).

### Whole-Exome Sequencing

More comprehensive methods such as WES and WGS identify high numbers of pharmacogenetic biomarkers. In addition, these sequencing approaches may facilitate the discovery of novel loci. While it is possible to reuse WES for PGx purposes for known variants, the application for novel variants is challenging as the investigators would need a confirmative study or extensive *in-vitro* research to attribute potential, newly identified variants in a particular gene to drug response. This is particularly true if it is not clear what functional effect the genetic variation exerts on protein function and/or expression. Van der Lee et al. investigated the feasibility of repurposing WES data for the extraction of a PGx panel of 42 variants in 11 pharmacogenes to provide a pharmacogenomic profile. Based on the Ubiquitous Pharmacogenomics (U-PGx; www.upgx.eu) panel which includes all the actionable genes and variants in the DPWG guidelines, the authors successfully extracted information regarding 39 variants out of the total 42. At least one actionable phenotype was present in 86% of the analyzed data from the included subjects. Although structural variants (SVs) and copy numbers in some pharmacogenes as well as CYP2C19, UGT1A1, CYP3A5, and CYP2D6 were not detected, and the study suffered from a small number of drug-related genes and a limited sample size, the authors concluded that the WES data can yield meaningful pharmacogenetic profiles for 7 out of 11 important pharmacogenes (van der Lee et al., 2020b). To assess the potential benefits and the limitations of using the clinical WES data for PGx analysis as a secondary finding, Cousin et al. analyzed the clinical WES data for the detection of any FGVs in three important pharmacogenes. PGx variants were extracted from the WES test results of patients and used in addition to their medical history data. A pharmacist interpreted the PGx data based on multiple resources including CPIC, UpToDate, Micromedex, and AskMayoExpert and used the information to perform a genotype-informed medication review. The authors concluded that PGx testing early in life would be helpful for prescribing physicians to make future prescribing decisions (Cousin et al., 2017). The accuracy and the concordance rate for the WES variant calling were also investigated by Wee Chua et al. The researchers performed a cross-comparison between the WES and MiSeq amplicon sequencing data in addition to the WES and iPLEX ADME PGx panel in 36 and 12 samples, respectively. The rate obtained for both comparisons was high (99%), which indicates that WES is a promising tool in PGx profiling of individuals with an estimated error rate of <1% (Chua et al., 2016). However, despite these positive results, an important limitation of WES is that several important PGx variants, including CYP2C19*17 and VKORC1, are located outside of the captured regions of routine whole-exome sequencing.

### Whole-Genome Sequencing

Complete genomic variants (including PGx-related markers) for an individual would be available through the utilization of the WGS approach. Although the big data interpretation of such tests is still challenging, a decrease in sequencing costs alongside the comprehensiveness of WGS may result in the method becoming a standard platform for clinical PGx tests.

Through using the WGS data from phase 1 of the 1,000 Genomes project and subsequent annotation, 69,319 variants including SNVs (94%) and InDels (6%) were revealed in 160 pharmacogenes (127 CPIC genes and 64 VIP genes from PharmGKB). Minor allele frequency for the variants was >1%, of which 8,207 were in strong linkage disequilibrium (LD) (*r* ^2^ > 0.8) with known PGx variants. The alterations were distributed in various parts of the genome including intronic, coding, and 5′ upstream and 3′ downstream regions. In the end, the authors identified putatively functional variants within known pharmacogenomic loci underlying drug response phenotypes and suggested direct testing instead of relying on LD, which is going to be different among populations. A limited sample size and exclusion of rare variants (MAF <0.01) in addition to a lack of an experimental validation study were reported as the main limitations of the investigation. However, the results from such PGx studies facilitate the translation of the findings of the genomic analysis into clinical practice (Choi et al., 2019). While the known PGx gene panels could be included in the WGS data and considered a source for clinical PGx and drug prescribing, the remainder of the information could still be useful for discovery studies.

The functional CNVs in ADME genes are distributed with significantly different frequencies across diverse populations (He et al., 2011; Martis et al., 2013). The NGS data could also be used for CNV calling in different ethnic backgrounds. The investigators used the integrated WGS and WES data from 1,000 Genome and ExAC repositories for CNV identification in 208 pharmacogenes. Novel CNVs (deletion in 84% and duplications in 91% of genes) across six different populations of non-Finnish Europeans, Africans, Finns, East Asians, South Asians, and admixed Americans were decoded successfully. The final result highlighted the necessity for the comprehensive NGS-based genotyping of the pharmacogenes for the CNV identification alongside their allele frequencies. The assessment of the contribution of such CNVs to the drug response outcomes is also possible through a population-specific analysis of rare variants (Santos et al., 2018). Applying NGS for recognizing the actionable variants in genomic profiles may lead to lifetime utilization of PGx information for related individuals. Furthermore, future bioinformatics tools could potentially be utilized for the NGS data re-analysis and the functional prediction of novel variants (Cousin et al., 2017).

As demonstrated, the targeted sequencing approaches are most suitable for genotyping of known PGx genes, including the low-frequency variants. For the discovery of novel pharmacogenes of interest, WGS and WES are considered better choices (Reisberg et al., 2019). WES and WGS also offer the possibility of data repurposing, which means that the clinicians can benefit from the existing clinical sequencing data to extract a PGx profile to inform drug treatment. Although the NGS data from different platforms offer many potential benefits, there are still several challenges and limitations which are discussed in the following sections.

## Challenges in the Application of NGS Platforms for the Decoding of PGx Variants in Specific Pharmacogenes

From the studies presented above, it appears that most types of variants in the coding and non-coding or regulatory parts of drug-related genes including SNVs, InDels, CNVs, and some structural alterations such as tandem substitutions could be identified with NGS, particularly with long-read sequencers and WGS. However, some well-known clinically actionable pharmacogenetic variants still pose a challenge for the NGS methods. Challenging genes include some core ADME genes, such as CYP2D6 which contains many different known (>100 * alleles, www.pharmvar.org) variants in different populations. Moreover, high sequence similarity and genetic recombination between real genes and close pseudogenes, such as CYP2D7 and CYP2D8, structural rearrangement complexities, and high CNVs among individuals present substantial challenges. Here, the routine short-read NGS approaches will not clarify the genetic profile of an individual and offer proper phenotype prediction. Furthermore, difficulties in the alignment procedures make interpretation and translation into clinical use complicated. Although some of these problems can be resolved by high-resolution techniques, including long-read sequencing, such sequencers with lower error rates (as well as PacBio Sequel HiFi II) are only available through highly specialized centers and are not yet applied in routine clinical practice (Yang et al., 2017). In addition, the technology is currently not being considered for the large-scale genome analysis in the PGx studies (van der Lee et al., 2020a).

Another example of a challenging pharmacogene is UGT1A1, with some important variants in the non-coding parts of the gene (TA repeats in the promoter of the gene, particularly UGT1A1*28, which affect the gene transcription and hence enzyme activity) (Bosma et al., 1995; Dalén et al., 1998; Numanagić et al., 2015). The gene harbors more than 113 functionally relevant variants, most of which reduce or enhance enzyme function, in addition to many other variants with unknown significance. The allele frequency is heavily population-specific, too. However, most of the panels focus on commonly known genotypes and could easily miss predictive variants in particular cases. By way of illustration, FDA approved the test for *28 allele but not *6 allele for irinotecan, although the latter is the main cause of the altered activity of the UGT1A enzyme in the Asian populations (Ikediobi et al., 2009). Also, the utilization of more comprehensive platforms such as WES is accompanied by poor and insufficient coverage for non-coding parts, which may result in the lower concordance and weak diplotype and CNV calls for the UGT1A1 gene (van der Lee et al., 2020b).

A third challenging region is the HLA genes. They are characterized by high sequence homology and prone to error in the capturing procedure and possible misalignment in the mapping processes. In addition, more than 21,000 known alleles and several pseudogenes and some InDels in the intronic regions of HLA class I and class II genes require the utilization of a proper platform, and more advanced IT infrastructure for the bioinformatics analysis and the identification of various potential predictive PGx markers, particularly in the newly studied populations (Klasberg et al., 2019). HLA alleles are important not only in PGx but also in other medical fields, including the genomic evaluation of multifactorial disorders and organ transplantation. Unfortunately, most of the HLA variants are rare and population-specific and are not included in routine clinical PGx testing (Nakkam et al., 2018). Today, many bioinformatics tools and algorithms available for HLA variant calling and haplotype phasing based on the WGS, WES, and targeted sequencing results. However, the high coverage of the genomic region is preferred as input for the allelic imputation by most software (Karnes et al., 2017). The available tools and their pros and cons have been discussed comprehensively in the literature (Ka et al., 2017; Kawaguchi et al., 2017; Xie et al., 2017). In general, to overcome the challenges of decoding PGx variants in specific genes, up-to-date knowledge of PGx-related genomics for physicians requesting the test in addition to the selection and utilization of an appropriate platform and interpretation tools for each situation by PGx test centers is required. This may also include previous knowledge of some particular PGx alleles with substrate-specific effects. For example, CYP2D6*17 encodes an enzyme with an increased capacity to metabolize haloperidol but an impaired ability to metabolize codeine (Oscarson et al., 1997; Wennerholm et al., 2002). In addition, occasional discrepancies between guidelines on the classification of genotypes into metabolic groups (which is key to formulating corresponding therapeutic recommendations) must also be considered (Caudle et al., 2020). Table 2 summarizes some challenging pharmacogenes and their main features that need to be taken into consideration during sequencing or panel design.

**TABLE 2:** Pharmacogenes with the associated challenges that render them difficult to genotype.

Gene	Challenge(s)	Reference
CYP2D6	–Structural variants and gene rearrangements	Taylor et al. (2020)
	–Pseudogenes	PharmVar structural variations CYP2D6
	–Copy Number Variations	
	–Presence of novel variants	
	–Highly polymorphic region	
	–Substrate-specific effects of some alleles	
UGT1A1	–Rare population-specific variants	Barbarino et al. (2014)
	–Variants in non-coding parts of the gene	Marques and Ikediobi, 2010
	–Independent haplotypes with less linkage disequilibrium	
VKORC1	–Important variants in non-coding parts of the gene	Saminathan et al. (2010)
		Owen et al., 2010
HLA	–Rare population-specific variants	Illing et al. (2017)
	–Highly polymorphic regions	Klasberg et al., 2019
SLC6A4	–Rare population-specific variants	Lam (2013)

## Challenges and Opportunities for Data Acquisition and Interpretation

The NGS data annotation, in the form of PGx phenotype prediction, is a highly specialized task that requires both molecular knowledge and clinical knowledge. The extraction of actionable, putative, or likely pathogenic variants from large, sophisticated raw data requires considerable time and effort as well as accurate validation methods. The current approaches include newly developed PGx dedicated tools for star allele calling in pharmacogenes (discussed in the following sections). Here, we address the key considerations, discuss some features of the common PGx-related tools, and propose solutions for managing the challenges.

### Targeted Sequencing Panels

Unlike with other genotyping approaches, performing a sequencing run always offers the possibility of decoding novel variants in the sequenced part(s). This has also been observed in the targeted sequencing panels of known pharmacogenes, where novel variations appeared in addition to common markers (Gulilat et al., 2019). Indeed, the variants with unknown clinical significance (VUS) in the NGS data and with no clear connection to pharmacogenetics present a real challenge as far as the implementation of such technologies in clinical practice is concerned. Nevertheless, handling VUS as potentially important identified variants is essential since if appropriate approaches to the correct interpretation were not available, the real functional alleles might simply be introduced as non-actionable. Therefore, a prediction is not feasible easily on the functionality of VUS to interpret the potential effects on the drug responses in a patient. However, because of the lower number of such findings in panels, replication and validation studies using other orthogonal genotyping methods, in silico algorithms, genetic screening for first degree relatives of the proband, and use of GWAS, HapMap, or gnomAD datasets for meta-analysis will be faster and more easier with regard to predicting and confirming the negative or neutral functionality of variants and demonstrating the phenotype associations in the targeted sequencing approaches (Svidnicki et al., 2020).

### Whole-Exome and Whole-Genome Sequencing

As expected, VUS are more common in WES and WGS. The situation becomes even more complicated when the results involve novel PGx genes. Online tools such as SIFT and PolyPhen2 as well as other algorithms, including CADD and PROVEAN, plus Ensembl based sources with multiple integrated tools like VEP and REVEL, are available for the prediction of the damaging effects of a large number of variants. However, these tools rely primarily on evolutionary conservation and utilize amino acid or nucleotide sequence alignment, which is less applicable to pharmacogenes. Also, low predictive value of these tools has recently been demonstrated (Lee et al., 2019; Zhou et al., 2019).

Furthermore, incidental findings (IFs), referred to as secondary findings in the ACMG recommendations (Kalia et al., 2017), can be expected in different types of high throughput sequencing and genotype screening methods. They are mostly defined as annotated functional variants in major drug-related genes which were not expected in the specified assessment but may be either related or unrelated to the particular medication taken by the patient. This adds to the complexity of reporting findings from PGx profiling, where the DNA variants may alter the drug efficacy or increase the risk of serious adverse drug reactions. Such findings could be reported as variants with potential usage in guiding therapy if they are managed properly through appropriate clinical genomic assays, vigorous genotype-phenotype correlation studies, and utilization of PGx-related sources for data interpretation and variant scoring (Lee et al., 2016). However, the existence of secondary findings would also be associated with some technical issues in the employed NGS platform. These issues include the percentage of coverage and type of sequencing methods as well as the number of evaluated individuals, evaluation of family members or randomly selected patients (Westbrook et al., 2013). Yet, not all secondary findings that are identified need to be reported in the result of a clinical test. The ACMG also declared a policy statement for reporting particular secondary findings in the clinical setting (L Blackburn et al., 2015; Miller et al., 2021). However, the statement is related to non-PGx secondary findings. Moreover, many pharmacogenetic variants are not disease-causing. Therefore, the relevance of reporting secondary findings may not be obvious at the time of submitting the report, particularly when only a specific set of pharmacogenes is tested. For the pharmacogenes connected with disease risk, the secondary findings may be handled in accordance with the current ACMG recommendations; that is, it is not necessary to provide a separate set of recommendations for those genes. Nevertheless, while the purpose of PGx testing is to exhaustively (and pre-emptively) profile genes that may potentially alter the drug response, curating and storing the information relevant to the future drug therapy may indicate that no findings should be considered “secondary,” particularly when untargeted methods as well as WES and WGS are employed.

### Recently Developed Bioinformatics Algorithms for PGx Variant Calling

Concentrated efforts have been undertaken to design and develop specific PGx tools for the identification of SNVs, CNVs, structural rearrangements, gene deletion, gene duplication/multiplication, haplotype phasing, diplotype calling, and phenotype prediction out of the NGS data in the clinical setting. The tools as well as Stargazer, PharmCAT, Astrolabe, Aldy, Cypripi, include special algorithms, which were designed for the interpretation of the PGx variants (Numanagić et al., 2015; Twist et al., 2016; Klein and Ritchie, 2018; Numanagić et al., 2018; Lee et al., 2019). Furthermore, some other tools including g-Nomic and PHARMIP were developed for providing recommendations based on the general information obtained from a PGx test (Sabater et al., 2019; Zidan et al., 2020). The advantages and the disadvantages of each of the tools have been demonstrated previously in the literature (Twesigomwe et al., 2020). Table 3 provides a concise overview of the key features of these tools. Stargazer, Astrolabe, and Aldy have been fully analyzed and are widely used in the field. Twesigomwe and colleagues have recently performed a comprehensive and systematic comparison of the functions of these three tools in calling different CYP2D6 variants. The results of the study demonstrate that Aldy and Astrolabe are better common and rare SNV callers compared to Stargazer. Yet, Stargazer outperformed the other tools in rare homozygous allele phasing due to its in-built supplementary algorithm. Calling InDel star alleles in the short-read NGS data and the hybrid rearrangements was challenging for all three algorithms. For other structural variants, gene deletion, duplication, and multiplications, Aldy demonstrated higher concordance in comparison to Stargazer and Astrolabe, respectively. Noticeably, Astrolabe performed weak structural variant calling in comparison to the other two tools. Although Stargazer displayed better performance in CNV calling and the identification of hybrid rearrangements, it simultaneously revealed the highest number of non-genotyped diplotypes for the samples including structural variants. Unfortunately, all three tools had difficulty calling diplotypes with high copy numbers. While these genotypes are very rare, they may still be considered an important variant in some isolated populations. The phenotype prediction and the clinical accuracy of Aldy, Astrolabe, and Stargazer were also evaluated. Remarkably, the concordances were higher than the diplotype concordances as the activity scoring systems may assign the same values as the true function of the wrongly genotyped samples. The impact of the sequencing coverage and the misalignment of InDels on genotyping accuracy was also investigated. The study, however, had some limitations. It used simulated data for most rare and structural variants, did not compare the performances of the three tools across the NGS data from the targeted custom-capture panels, and did not compare the impacts of different aligners on the variant calling processes. Novel SNVs calling was also not analyzed in the study and reliable validation studies were not included (Twesigomwe et al., 2020). Aldy and Stargazer may also result in false-positive/false-negative results in small variant calling, since they rely on initial read alignments. Another major obstacle is that two of the three tools does not support the GRCh38 genome assembly and that the investigators may need to lift their alignments to GRCh37 (i.e., https://genome.ucsc.edu/cgi-bin/hgLiftOver). To address these challenges, Chen et al. developed Cyrius, a novel bioinformatics method for all classes of variants and haplotype calling from CYP2D6 in the WGS data (also included in Table 3). The tool can overcome CYP2D6 and CYP2D7 homology challenges and work with both GRCh37 and 38 to accurately genotype CYP2D6 with a higher overall concordance rate with true genotypes (99.3%). Compared to Aldy and Stargazer, superior genotyping was demonstrated for both GeT-RM and long-read data, and the application of the method led to improved understanding of CYP2D6 genetic diversity within five ethnic groups. The authors are currently extending the method to genotype other pharmacogenes with a paralog, CYP2A6 and CYP2B6, and plan to apply it to more genes in the future (Chen et al., 2021). Overall, it is useful to be aware of the specifications and the features of each of the tools in order to increase their utility while applying such algorithms to calling different PGx variants out of high throughput sequencing results.

**TABLE 3:** Key features of the PGx dedicated variant functional prediction tools.

Tool/Algorithm	Main features	Reference
Stargazer	Stargazer calls the star alleles from the NGS data by detecting SNVs, InDels, and structural variants. Stargazer detects variations with structural changes including gene duplications, deletions, and conversions by calculating the paralog-specific copy numbers from read depth	Lee et al. (2019)
PharmCAT	Pharmacogenomics Clinical Annotation Tool (PharmCAT) captures the variants indicated in guidelines from a genomic data set derived from sequencing or genotyping technologies (i.e., VCF), infers haplotypes and diplotypes, and generates a report containing genotype/diplotype-based annotations, as well as guidelines and recommendations according to CPIC guidelines	Sangkuhl et al. (2020)
Aldy	Aldy is a computational tool that performs allelic decomposition of highly polymorphic, multi-copy genes through the use of the whole or targeted genome sequencing data and identifies multiple rare and novel alleles for several important pharmacogenes	Numanagić et al. (2018)
Astrolabe	Astrolabe (former Constellation) is a computational method and probabilistic scoring system that enables automated ascertainment of CYP2D6 and CYP2D19 activity scores from the unphased NGS data, aligned with the catalog of pharmacogenetic alleles with high percentage of analytic sensitivity and specificity	Twist et al. (2017)
Cypripi	Cypripi is an algorithm that computationally assumes CYP2D6 genotype at base-pair resolution from the high throughput sequencing data. It can resolve complex genotypes, including the alleles that are the product of the duplication, deletion, and fusion events involving CYP2D6 and its related pseudogene, CYP2D7	Numanagić et al. (2015)
g-Nomic	g-Nomic is PGx interpretation software that provides recommendations on the suitability of a given combination of drugs for each patient according to their genes and polymedication	Sabater et al. (2019)
PHARMIP	PHARMIP uses drug modeled structure and up-to-date bioinformatics tools and/or databases to understand the genetic factors that cause drug-related adverse reactions	Zidan et al. (2020)
Cyrius	Superior, accurate genotyping of CYP2D6 compared to other existing methods as well as Aldy and Stargazer. All types of variants and haplotype calling in addition to the structural and homology analysis will be covered for both GRCh38 and 37 genome builds	Chen et al. (2021)

### Solutions for the Management of Challenges in Applying the NGS-PGx Tests in the Clinic

Here, we present three main problems which may arise during clinical NGS testing for PGx in everyday practice and discuss solutions.

Firstly, based on the type of panel or other selected approaches, the setup and the initiation of NGS tests (covering PGx markers) in every clinic will require a substantial investment and reimbursement by insurance companies, bioinformatics infrastructure, specific software and computational tools, and professional clinical experts for data interpretation. In addition, validation studies to determine and improve the clinical utility and the validity are essential. Once a positive evaluation has been performed by public and private payers, relevant NGS-derived PGx tests could be considered for implementation in routine clinical practice. Estimated costs of PGx profiling may vary substantially depending on the type of test applied. Is the PGx assessment a pre-emptive NGS test or repurposed findings from diagnostic WES/WGS? Currently, the test coverage and reimbursement are still considered major barriers to routine clinical use. Enhancing physicians’ awareness of the type of test to be requested, gaining third-party support, increasing the number of clients through direct-to-consumer genetic testing companies, and decreasing the cost of tests due to advances in diagnostic technologies may play an essential role in bringing the clinical utility of PGx tests to the attention of insurance companies (L Rogers et al., 2020). While many related services are currently limited to reactive single-gene testing, some clinical centers offer routine pre-emptive PGx tests. For example, all patients treated for an active disease at St. Jude Research Hospital are offered PGx testing (www.stjude.org/pg4kds). Recently, Anderson et al. performed a large-scale study in the United States and demonstrated that only a few core pharmacogenes, including CYP2C19, CYP2D6, CYP2C9, VKORC1, UGT1A1, and HLA class I, were covered by the patients’ insurance (Anderson et al., 2020).

Secondly, as mentioned previously, the evolutionary conservation is less applicable to the drug-related genes and therefore the conventional computational algorithms have low predictive accuracy when applied to the pharmacogenetic variants. The difficulties with novel and big data interpretation could be overcome by applying combined and optimized calculation tools and algorithms (at least 6-7 of such bioinformatics tools) for allele imputation (see Appendix 1) of PGx single- or multi-marker signatures, as well as confirming such genetic variants as predictive for the drug response with more accuracy (Zhou et al., 2019; Tafazoli et al., 2021). However, not all pharmacogenes have this limitation. Indeed, some genes appear relatively free of evolutionary constraints and are highly similar to other genes. This is particularly true for the genes that are involved in the transfer of endogenous substances (i.e., OTC1). Whenever a novel PGx variant is identified in evolutionarily conserved positions, such genes may still benefit from routine predictor tools to indicate their functional impact (Shu et al., 2003). However, in the absence of distinct clinical data, both computational and laboratory models are needed for the genotype-guided drug therapy based on previously unreported genomic variants (Shrestha et al., 2018).

Other PGx specific computational models and algorithms with a high sensitivity and specificity have also been developed for the prediction of the loss of function and/or the functionally neutral variations. The scores obtained with the models could provide quantitative estimation of the impact of different variants on the gene function. A comprehensive analysis of the computational prediction methods and evaluation of the recent progress in the functional interpretation of non-coding variants for drug-metabolizing enzymes and transporters is provided by Zhou and colleagues (Zhou et al., 2018). Once the functionality of a variant is known, the effect on drug pharmacology needs to be estimated. For this, pathway analysis databases as well as DAVID, Human Metabolome Database, String-db, and KEGG could be used to identify the molecular connections between the altered allele(s) in specific genes and the other related genes in the cell. Moreover, newly developed PGx specific tools such as Aldy, Stargazer, Astrolabe, and Cyrius can also help with NGS data processing in the PGx analysis (Klein and Ritchie, 2018; Lee et al., 2019). Table 4 lists some databases which are useful in interpreting the results of the clinical PGx analysis. We have also recently reviewed the software and the algorithms dedicated to the functional prediction alongside the related mechanism of action in such tools while using the PGx functional analysis (Tafazoli et al., 2021). After finding a potentially strong relationship between the identified variant(s) and the drug response, particular *in-vitro* assessments as well as cell line modifications may be considered for exploring the functional consequences of the altered alleles and diplotypes on the activity of the related protein. However, the latter is not appropriate in clinical use as it increases the turnaround time considerably. As the final step, the clinical association analysis will confirm the connection between the novel variants and the drug response phenotypes in the patients. Needless to say, it is suitable solely for the patient data analysis and not pre-emptive PGx profiling of a healthy individual with no clinically observable phenotype (Ji et al., 2013).

**TABLE 4:** Useful databases for PGx results interpretation in the clinical practice.

Database	Main Activities and Features	Link	Reference
PharmGKB	The Pharmacogenomics Knowledgebase is a truly comprehensive and publicly available, online knowledgebase responsible for the aggregation, curation, integration, and dissemination of the knowledge regarding the impact of the human genetic variation on the drug response	https://www.pharmgkb.org/index.jsp	Barbarino et al. (2018)
CPIC	The Clinical Pharmacogenetics Implementation Consortium (CPIC^®^) is an international consortium to address the clinical implementation of the pharmacogenetic tests by creating, curating, and posting freely available, peer-reviewed, evidence-based, updatable, and detailed gene/drug clinical practice guidelines	https://cpicpgx.org/	Relling and Klein (2011)
DPWG	The Dutch Pharmacogenetics Working Group includes clinical pharmacists, physicians, clinical pharmacologists, clinical chemists, epidemiologists, and toxicologists to develop pharmacogenetics-based therapeutic (dose) recommendations and assist the drug prescribers and the pharmacists by integrating the recommendations into computerized systems for drug prescription and automated medication surveillance	https://www.pharmgkb.org/page/dpwg	JJ Swen et al. (2011)
PharmVar	The Pharmacogene Variation (PharmVar) Consortium is a central repository for the pharmacogene (PGx) variation that focuses on the haplotype structure and the allelic variation. The information in this resource facilitates the interpretation of the pharmacogenetic test results to guide the precision medicine	https://www.pharmvar.org/	Gaedigk et al. (2018)
PMKB	The Precision Medicine Knowledgebase (PMKB) is a project of the Institute of Precision Medicine (IPM) at Weill Cornell Medicine, which is organized to provide information about the clinical cancer variants and the interpretations in a structured way as well as allowing the users to submit and edit the existing entries for the continued growth of the knowledgebase. All changes are reviewed by cancer pathologists	https://pmkb.weill.cornell.edu	Huang et al. (2017)
PharmaADME	An industry-initiated effort launched to develop a consensus, “Core List” of standardized “evidence-based” drug metabolizing (ADME) genetic biomarkers that are broadly applicable to many pharmaceutical clinical trials and FDA drug submissions	http://www.pharmaadme.org/joomla/	pharmaadme and www.pharmaadme.org
Flockhart Table	The website provides a table designed as a hypothesis testing, teaching, and reference tool for the physicians and researchers interested in the drug interactions that are the result of the competition for or effects on the human cytochrome P450 system. The table contains lists of drugs in columns under the designation of specific cytochrome P450 isoforms	https://drug-interactions.medicine.iu.edu/MainTable.aspx	Flockhart and Oesterheld (2000)
SEAPharm	The Southeast Asian Pharmacogenomics Research Network (SEAPharm) established in Asia to enable and strengthen the PGx research among various PGx communities within but not limited to countries in SEA, with the ultimate goal of supporting PGx implementation in the region	–	Chumnumwat et al. (2019)
PGRN	The Pharmacogenomics Research Network, PGRN I–III, was funded from 2000 through 2015 by multiple Institutes and Centers of the NIH. The network catalyzed pharmacogenomics discoveries both nationally and internationally through the conduct of collaborative research focused on the discovery and the translation of the the genetic determinants of the drug response, to enable safer and more effective drug therapies	https://www.pgrn.org/	–
SuperCYP	A comprehensive database on cytochrome P450 enzymes including a tool for analysis of the CYP-drug interactions	https://bioinformatics.charite.de/supercyp/	Preissner et al. (2010)
FDA-Pharmacogenomic	Table of pharmacogenomic biomarkers in drug labeling	https://www.fda.gov/drugs/science-and-research-drugs/table-pharmacogenomic-biomarkers-drug-labeling	–

Finally, while well-known and annotated PGx variant(s) can be used immediately in patient care, the clinical translation and utilization of newly introduced variants requires substantial evidence and records of gene-drug interaction as well as phenotyping data. Nevertheless, such data would be stored primarily for the research purposes and the patient may be recontacted for further investigations. Since the prediction of an individual’s metabolic status is very important for drug dosage modifications in a clinic, the translation of the sequencing results into phenotype assignment must follow the universal standardized test interpretation approaches. A gene continuum activity score system has been introduced to deal with such situations and may be accepted by reference laboratories and medical centers for converting the genotype data to the clinically actionable recommendations (Hicks et al., 2014). However, to facilitate the incorporation of the high throughput derived PGx reports in the clinical setting, it is necessary to provide the healthcare professionals with more applicable, evidence-based results and employ standardized and updated cohort and case reports (Giri et al., 2019; Krebs and Milani, 2019).

## Conclusion

The NGS technologies have been used in the PGx research studies for a decade. The rapid development in accessories and supporting bioinformatics tools in addition to the reduced cost and the technological advancement that will allow for testing of a larger number of drug-related genes and biomarkers will result in the widespread use of such methods in various clinical settings. The main challenges are management of identified VUS, a lack of specific variant caller software, poor haplotype phasing, insufficient coverage of some parts of the genome by different platforms, limited capacity to assess variant functionality *in-vitro*, and limited ability to assess functionality through computational approaches. Nevertheless, the application of NGS in PGx testing in the clinical practice is continually increasing, paving the way for new PGx variant discovery and a bright future for pharmacogenomics-guided drug treatment.

## Abbreviations

ACMG, American College of Medical Genetics and Genomics; CADD, Combined Annotation-Dependent Depletion; CAP, College of American Pathologists; CLIA, Clinical Laboratory Improvement Amendments; CNV, Copy Number Variation; CYP, cytochrome P450; ExAC, Exome Aggregation Consortium; FDA, US Food and Drug Administration; GWAS, Genome-Wide Association Study; InDel, Insertion/Deletion; MAF, minor allele frequency; PROVEAN, Protein Variation Effect Analyzer; REVEL, Rare Exome Variant Ensemble Learner; SNP, Single Nucleotide Polymorphism; SNV, Single Nucleotide Variation; SV, structural variant; VCF, variant calling format; VEP, Variant Effect Predictor; VIP, Very Important Pharmacogenes.

## Appendix 1: mini-glossary of the NGS terminologies used in this article

**Table:**

Targeted sequencing	Sequencing of specific parts of the genome or sets of genes (multiple genes) at once
Whole-exome sequencing	Sequencing of all exonic (protein-coding) regions of the genome. It also includes some important flanking sequences
Whole-genome sequencing	Sequencing of the entire genome of an organism, including all the non-coding and coding parts in addition to the mitochondrial DNAs. It is the most comprehensive sequencing method among others
Long-read sequencing	Newer sequencing methods (also called third-generation technologies) with the ability to read and produce long sequences of DNA between 10,000 and 100,000 base pairs at one runtime. The method is useful, particularly for the structural variant detection and haplotype phasing
Gene panel	A specific set of genes selected for particular analysis purposes as well as sequencing methods or disease-specific gene profiling
PGx panel	A specific set of pharmacogenes or drug target genes selected for particular analysis purposes
Coverage	The number of times a portion of the genome is sequenced in a sequencing reaction. Frequently expressed as “depth of coverage” and numerically as 1X, 2X, 3X, etc.
Depth of coverage	See above
VCF file	Variant calling format is a standard variant reporting format which was invented during the 1,000 Genomes project. Such files display the genomic variants with their coordinates in the NGS results
Secondary findings	Unrelated genomic variants to the primary purpose of the test revealed during a sequencing run
Haplotype phasing	Determination of paternal or maternal origins (inheritance) of each chromosome while putting into haplotypes. In this way, the researchers can assign the alleles to the paternal and maternal chromosomes and obtain a comprehensive picture of genomic variants for the specific haplotype
	Statistical estimation of the haplotypes from the genotyping data is also called haplotype phasing
Allele imputation	A statistical method for inferring the genotypes that are not directly measured. Estimation of unobserved genotype, including genetic markers from known haplotype or reference genotype. Particularly beneficial in GWAS studies
VUS	A genetic variant for which the association with a specific phenotype cannot be determined definitively

## References

[B1] Ammar R. Paton T. A. Torti D. Shlien A. Bader G. D. (2015). Long Read Nanopore Sequencing for Detection of HLA and CYP2D6 Variants and Haplotypes. F1000Res 4, 17. 10.12688/f1000research.6037.2 25901276 PMC4392832

[B2] Anderson H. D. Crooks K. R. Kao D. P. Aquilante C. L. (2020). The Landscape of Pharmacogenetic Testing in a US Managed Care Population. Genet. Med. 22, 1247–1253. 10.1038/s41436-020-0788-3. 32291400 PMC7332417

[B3] Arbitrio M. Di Martino M. Scionti F. Agapito G. Hiram Guzzi P. Cannataro M. (2016). DMETTM (Drug Metabolism Enzymes and Transporters): a Pharmacogenomic Platform for Precision Medicine. Oncotarget 5 (33), 54028–54050. 10.18632/oncotarget.9927 PMC5288240 27304055

[B4] Ardui S. Race V. Zablotskaya A. Hestand M. S. Van Esch H. Devriendt K. (2017). Detecting AGG Interruptions in Male and Female FMR1 Premutation Carriers by Single-Molecule Sequencing. Hum. Mutat. 38, 324–331. 10.1002/humu.23150 27883256

[B5] Barbarino J. M. Haidar C. E. Klein T. E. Altman R. B. (2014). PharmGKB Summary. Pharmacogenetics and genomics 24, 177–183. 10.1097/FPC.0000000000000024 24492252 PMC4091838

[B6] Barbarino J. M. Whirl-Carrillo M. Altman R. B. Klein T. E. (2018). PharmGKB: a Worldwide Resource for Pharmacogenomic Information. Wires Syst. Biol. Med. 10, e1417. 10.1002/wsbm.1417 PMC6002921 29474005

[B7] Bielinski S. J. Olson J. E. Pathak J. Weinshilboum R. M. Wang L. Lyke K. J. (2014). Preemptive Genotyping for Personalized Medicine: Design of the Right Drug, Right Dose, Right Time-Using Genomic Data to Individualize Treatment Protocol. Mayo Clinic Proc. 89, 25–33. 10.1016/j.mayocp.2013.10.021 PMC3932754 24388019

[B8] Bosma P. J. Chowdhury J. R. Bakker C. Gantla S. De Boer A. Oostra B. A. (1995). The Genetic Basis of the Reduced Expression of Bilirubin UDP-Glucuronosyltransferase 1 in Gilbert's Syndrome. N. Engl. J. Med. 333, 1171–1175. 10.1056/NEJM199511023331802 7565971

[B9] Caspar S. M. Schneider T. Meienberg J. Matyas G. (2020). Added Value of Clinical Sequencing: WGS-Based Profiling of Pharmacogenes. Ijms 21, 2308. 10.3390/ijms21072308 PMC7178228 32225115

[B10] Caudle K. E. Sangkuhl K. Whirl‐Carrillo M. Swen J. J. Haidar C. E. Klein T. E. (2020). Standardizing CYP 2D6 Genotype to Phenotype Translation: Consensus Recommendations from the Clinical Pharmacogenetics Implementation Consortium and Dutch Pharmacogenetics Working Group. Clin. Transl Sci. 13, 116–124. 10.1111/cts.12692 31647186 PMC6951851

[B11] Chen X. Shen F. Gonzaludo N. Malhotra A. Rogert C. Taft R. J. (2021). Cyrius: Accurate CYP2D6 Genotyping Using Whole-Genome Sequencing Data. Pharmacogenomics J. 21, 251–261. 10.1038/s41397-020-00205-5 33462347 PMC7997805

[B12] Choi J. Tantisira K. G. Duan Q. L. (2019). Whole Genome Sequencing Identifies High-Impact Variants in Well-Known Pharmacogenomic Genes. Pharmacogenomics J. 19, 127–135. 10.1038/s41397-018-0048-y 30214008 PMC6417988

[B13] Chua E. W. Cree S. L. Ton K. N. T. Lehnert K. Shepherd P. Helsby N. (2016). Cross-Comparison of Exome Analysis, Next-Generation Sequencing of Amplicons, and the iPLEX ADME PGx Panel for Pharmacogenomic Profiling. Front. Pharmacol. 7, 1. 10.3389/fphar.2016.00001 26858644 PMC4726781

[B14] Chumnumwat S. Lu Z. H. Sukasem C. Winther M. D. Capule F. R. Abdul Hamid A. A. a. t. (2019). Southeast Asian Pharmacogenomics Research Network (SEAPharm): Current Status and Perspectives. Public health genomics 22, 132–139. 10.1159/000502916 31587001

[B15] Cohn I. Paton T. A. Marshall C. R. Basran R. Stavropoulos D. J. Ray P. N. (2017). Genome Sequencing as a Platform for Pharmacogenetic Genotyping: a Pediatric Cohort Study. Npj Genomic Med. 2, 19. 10.1038/s41525-017-0021-8 PMC5677914 29263831

[B16] Cousin M. A. Matey E. T. Blackburn P. R. Boczek N. J. Mcallister T. M. Kruisselbrink T. M. (2017). Pharmacogenomic Findings from Clinical Whole Exome Sequencing of Diagnostic Odyssey Patients. Mol. Genet. Genomic Med. 5, 269–279. 10.1002/mgg3.283 28546997 PMC5441410

[B17] Cruz-Correa O. F. León-Cachón R. B. R. Barrera-Saldaña H. A. Soberón X. (2017). Prediction of Atorvastatin Plasmatic Concentrations in Healthy Volunteers Using Integrated Pharmacogenetics Sequencing. Pharmacogenomics 18, 121–131. 10.2217/pgs-2016-0072 27976987

[B18] Dalén P. Dahl M.-L. Ruiz M. L. B. Nordin J. Bertilsson L. (1998). 10-hydroxylation of Nortriptyline in white Persons with 0, 1, 2, 3, and 13 Functional CYP2D6 Genes*. Clin. Pharmacol. Ther. 63, 444–452. 10.1016/S0009-9236(98)90040-6 9585799

[B19] Dorado P. Cáceres M. C. Pozo-Guisado E. Wong M.-L. Licinio J. Llerena A. (2005). Development of a PCR-Based Strategy forCYP2D6genotyping Including Gene Multiplication of Worldwide Potential Use. Biotechniques 39, S571–S574. 10.2144/000112044 18957039

[B20] Flockhart D. A. Oesterheld J. R. (2000). Cytochrome P450-Mediated Drug Interactions. Child. Adolescent Psychiatric Clinics North. America 9, 43–76. 10.1016/s1056-4993(18)30135-4 10674190

[B21] Gaedigk A. Ingelman-Sundberg M. Miller N. A. Leeder J. S. Whirl-Carrillo M. Klein T. E. (2018). The Pharmacogene Variation (PharmVar) Consortium: Incorporation of the Human Cytochrome P450 (CYP ) Allele Nomenclature Database. Clin. Pharmacol. Ther. 103, 399–401. 10.1002/cpt.910 29134625 PMC5836850

[B22] Giri J. Moyer A. M. Bielinski S. J. Caraballo P. J. (2019). Concepts Driving Pharmacogenomics Implementation into Everyday Healthcare. Pgpm Vol. 12, 305–318. 10.2147/PGPM.S193185 PMC6826176 31802928

[B23] Gordon A. S. Fulton R. S. Qin X. Mardis E. R. Nickerson D. A. Scherer S. (2016). PGRNseq. Pharmacogenetics and genomics 26, 161–168. 10.1097/FPC.0000000000000202 26736087 PMC4935646

[B24] Guchelaar H.-J. (2018). Pharmacogenomics, a Novel Section in the European Journal of Human Genetics. Eur. J. Hum. Genet. 26, 1399–1400. 10.1038/s41431-018-0205-4 29967335 PMC6138632

[B25] Gulilat M. Lamb T. Teft W. A. Wang J. Dron J. S. Robinson J. F. (2019). Targeted Next Generation Sequencing as a Tool for Precision Medicine. BMC Med. genomics 12, 81. 10.1186/s12920-019-0527-2 31159795 PMC6547602

[B26] Han S. Park J. Lee J. Lee S. Kim H. Han H. (2017). Targeted Next-Generation Sequencing for Comprehensive Genetic Profiling of Pharmacogenes. Clin. Pharmacol. Ther. 101, 396–405. 10.1002/cpt.532 27727443

[B27] He Y. Hoskins J. M. Mcleod H. L. (2011). Copy Number Variants in Pharmacogenetic Genes. Trends Molecular Medicine 17, 244–251. 10.1016/j.molmed.2011.01.007 PMC3092840 21388883

[B28] Hicks J. Swen J. Gaedigk A. (2014). Challenges in CYP2D6 Phenotype Assignment from Genotype Data: a Critical Assessment and Call for Standardization. Cdm 15, 218–232. 10.2174/1389200215666140202215316 24524666

[B29] Hippman C. Nislow C. (2019). Pharmacogenomic Testing: Clinical Evidence and Implementation Challenges. Jpm 9, 40. 10.3390/jpm9030040 PMC6789586 31394823

[B30] Huang L. Fernandes H. Zia H. Tavassoli P. Rennert H. Pisapia D. (2017). The Cancer Precision Medicine Knowledge Base for Structured Clinical-Grade Mutations and Interpretations. J. Am. Med. Inform. Assoc. 24, 513–519. 10.1093/jamia/ocw148 27789569 PMC5391733

[B31] Ikediobi O. Shin J. Nussbaum R. Phillips K. Translational U. C. F. Medicine P. R. O. P. (2009). Addressing the Challenges of the Clinical Application of Pharmacogenetic Testing. Clin. Pharmacol. Ther. 86, 28–31. 10.1038/clpt.2009.30 19536122 PMC2910521

[B32] Illing P. T. Purcell A. W. Mccluskey J. (2017). The Role of HLA Genes in Pharmacogenomics: Unravelling HLA Associated Adverse Drug Reactions. Immunogenetics 69, 617–630. 10.1007/s00251-017-1007-5 28695285

[B33] Illumina (2020). Infinium® Global Screening Array-24 v3.0 BeadChip. Availableat: https://emea.illumina.com/content/dam/illumina-marketing/documents/products/datasheets/infinium-global-screening-array-data-sheet-370-2016-016.pdf

[B34] Ji Y. Biernacka J. M. Hebbring S. Chai Y. Jenkins G. D. Batzler A. (2013). Pharmacogenomics of Selective Serotonin Reuptake Inhibitor Treatment for Major Depressive Disorder: Genome-wide Associations and Functional Genomics. Pharmacogenomics J. 13, 456–463. 10.1038/tpj.2012.32 22907730 PMC3941038

[B35] Jiang M. You J. H. (2015). Review of Pharmacoeconomic Evaluation of Genotype-Guided Antiplatelet Therapy. Expert Opin. Pharmacother. 16, 771–779. 10.1517/14656566.2015.1013028 25660101

[B36] Johnson J. A. Burkley B. M. Langaee T. Y. Clare-Salzler M. J. Klein T. E. Altman R. B. (2012). Implementing Personalized Medicine: Development of a Cost-Effective Customized Pharmacogenetics Genotyping Array. Clin. Pharmacol. Ther. 92, 437–439. 10.1038/clpt.2012.125 22910441 PMC3454443

[B37] Ka S. Lee S. Hong J. Cho Y. Sung J. Kim H.-N. (2017). HLAscan: Genotyping of the HLA Region Using Next-Generation Sequencing Data. BMC bioinformatics 18, 1–11. 10.1186/s12859-017-1671-3 28499414 PMC5427585

[B38] Kalia S. S. Adelman K. Adelman K. Bale S. J. Chung W. K. Eng C. (2017). Recommendations for Reporting of Secondary Findings in Clinical Exome and Genome Sequencing, 2016 Update (ACMG SF v2.0): a Policy Statement of the American College of Medical Genetics and Genomics. Genet. Med. 19, 249–255. 10.1038/gim.2016.190 27854360

[B39] Karnes J. H. Shaffer C. M. Bastarache L. Gaudieri S. Glazer A. M. Steiner H. E. (2017). Comparison of HLA Allelic Imputation Programs. PLoS One 12, e0172444. 10.1371/journal.pone.0172444 28207879 PMC5312875

[B40] Kawaguchi S. Higasa K. Shimizu M. Yamada R. Matsuda F. (2017). HLA‐HD: An Accurate HLA Typing Algorithm for Next‐generation Sequencing Data. Hum. Mutat. 38, 788–797. 10.1002/humu.23230 28419628

[B41] Klasberg S. Surendranath V. Lange V. Schöfl G. (2019). Bioinformatics Strategies, Challenges, and Opportunities for Next Generation Sequencing-Based HLA Genotyping. Transfus. Med. Hemother 46, 312–325. 10.1159/000502487 31832057 PMC6876610

[B42] Klein K. Tremmel R. Winter S. Fehr S. Battke F. Scheurenbrand T. (2019). A New Panel-Based Next-Generation Sequencing Method for ADME Genes Reveals Novel Associations of Common and Rare Variants with Expression in a Human Liver Cohort. Front. Genet. 10, 7. 10.3389/fgene.2019.00007 30766545 PMC6365429

[B43] Klein T. E. Ritchie M. D. (2018). PharmCAT: a Pharmacogenomics Clinical Annotation Tool. Clin. Pharmacol. Ther. 104, 19–22. 10.1002/cpt.928 29194583 PMC5984125

[B44] Krebs K. Milani L. (2019). Translating Pharmacogenomics into Clinical Decisions: Do Not Let the Perfect Be the Enemy of the Good. Hum. Genomics 13, 1–13. 10.1186/s40246-019-0229-z 31455423 PMC6712791

[B45] L Rogers S. Keeling N. J. Giri J. Gonzaludo N. Jones J. S. Glogowski E. (2020). PARC Report: a Health-Systems Focus on Reimbursement and Patient Access to Pharmacogenomics Testing. Pharmacogenomics 21, 785–796. 10.2217/pgs-2019-0192 32748688

[B46] Lam Y. W. F. (2013). Scientific Challenges and Implementation Barriers to Translation of Pharmacogenomics in Clinical Practice. ISRN Pharmacol. 2013, 1–17. 10.1155/2013/641089 PMC3603526 23533802

[B47] Larsen J. B. Rasmussen J. B. (2017). Pharmacogenetic Testing Revisited: 5' Nuclease Real-Time Polymerase Chain Reaction Test Panels for Genotyping CYP2D6 and CYP2C19. Pharmgenomics Pers Med. 10, 115–128. 10.2147/PGPM.S131580 28458572 PMC5403119

[B48] L. Blackburn H. Schroeder B. Turner C. D. Shriver C. L. Ellsworth D. E. Ellsworth R. (2015). Management of Incidental Findings in the Era of Next-Generation Sequencing. Cg 16, 159–174. 10.2174/1389202916666150317232930 PMC4460220 26069456

[B49] Lee E. M. J. Xu K. Mosbrook E. Links A. Guzman J. Adams D. R. (2016). Pharmacogenomic Incidental Findings in 308 Families: The NIH Undiagnosed Diseases Program Experience. Genet. Med. 18, 1303–1307. 10.1038/gim.2016.47 27253732 PMC5133159

[B50] Lee S.-B. Wheeler M. M. Patterson K. Mcgee S. Dalton R. Woodahl E. L. (2019). Stargazer: a Software Tool for Calling star Alleles from Next-Generation Sequencing Data Using CYP2D6 as a Model. Genet. Med. 21, 361–372. 10.1038/s41436-018-0054-0 29875422 PMC6281872

[B51] Lemieux Perreault L.-P. Zaïd N. Cameron M. Mongrain I. Dubé M.-P. (2018). Pharmacogenetic Content of Commercial Genome-wide Genotyping Arrays. Pharmacogenomics 19, 1159–1167. 10.2217/pgs-2017-0129 30272537

[B52] Liau Y. Maggo S. Miller A. L. Pearson J. F. Kennedy M. A. Cree S. L. (2019). Nanopore Sequencing of the Pharmacogene CYP2D6 Allows Simultaneous Haplotyping and Detection of Duplications. Pharmacogenomics 20, 1033–1047. 10.1101/57628010.2217/pgs-2019-0080 31559921

[B53] Madian A. G. Wheeler H. E. Jones R. B. Dolan M. E. (2012). Relating Human Genetic Variation to Variation in Drug Responses. Trends Genetics 28, 487–495. 10.1016/j.tig.2012.06.008 PMC3448823 22840197

[B54] Mantere T. Kersten S. Hoischen A. (2019). Long-read Sequencing Emerging in Medical Genetics. Front. Genet. 10, 426. 10.3389/fgene.2019.00426 31134132 PMC6514244

[B55] Marques S. C. Ikediobi O. N. (2010). The Clinical Application of UGT1A1pharmacogenetic Testing: Gene-Environment Interactions. Hum. Genomics 4, 1–12. 10.1186/1479-7364-4-4-238 PMC3525209 20511137

[B56] Martis S. Mei H. Vijzelaar R. Edelmann L. Desnick R. J. Scott S. A. (2013). Multi-ethnic Cytochrome-P450 Copy Number Profiling: Novel Pharmacogenetic Alleles and Mechanism of Copy Number Variation Formation. Pharmacogenomics J. 13, 558–566. 10.1038/tpj.2012.48 23164804 PMC3580117

[B98] Miller D. T. Lee K. Chung W. K. Gordon A. S. Herman G. E. Klein T. E. (2021). ACMG SF v3. 0 List for Reporting of Secondary Findings in Clinical Exome and Genome Sequencing: a Policy Statement of the American College of Medical Genetics and Genomics (ACMG). Genet. Med., 1–10. 10.1038/s41436-021-01278-8 PMC13097145 34012068

[B57] Mizzi C. Peters B. Mitropoulou C. Mitropoulos K. Katsila T. Agarwal M. R. (2014). Personalized Pharmacogenomics Profiling Using Whole-Genome Sequencing. Pharmacogenomics 15, 1223–1234. 10.2217/pgs.14.102 25141897

[B58] Nakkam N. Konyoung P. Kanjanawart S. Saksit N. Kongpan T. Khaeso K. (2018). HLA Pharmacogenetic Markers of Drug Hypersensitivity in a Thai Population. Front. Genet. 9, 277. 10.3389/fgene.2018.00277 30127801 PMC6087736

[B59] Numanagić I. Malikić S. Pratt V. M. Skaar T. C. Flockhart D. A. Sahinalp S. C. (2015). Cypiripi: Exact Genotyping of CYP2D6 Using High-Throughput Sequencing Data. Bioinformatics 31, i27–34. 10.1093/bioinformatics/btv232 26072492 PMC4542776

[B60] Numanagić I. Malikić S. Ford M. Qin X. Toji L. Radovich M. (2018). Allelic Decomposition and Exact Genotyping of Highly Polymorphic and Structurally Variant Genes. Nat. Commun. 9, 1–11. 10.1038/s41467-018-03273-1 29483503 PMC5826927

[B61] Oscarson M. Hidestrand M. Johansson I. Ingelman-Sundberg M. (1997). A Combination of Mutations in the CYP2D6*17(CYP2D6Z) Allele Causes Alterations in Enzyme Function. Mol. Pharmacol. 52, 1034–1040. 10.1124/mol.52.6.1034 9415713

[B62] Owen R. P. Gong L. Sagreiya H. Klein T. E. Altman R. B. (2010). VKORC1 Pharmacogenomics Summary. Pharmacogenetics and genomics 20, 642–644. 10.1097/FPC.0b013e32833433b6 19940803 PMC3086043

[B63] Pharmaadme Www.Pharmaadme.Org .

[B64] Preissner S. Kroll K. Dunkel M. Senger C. Goldsobel G. Kuzman D. (2010). SuperCYP: a Comprehensive Database on Cytochrome P450 Enzymes Including a Tool for Analysis of CYP-Drug Interactions. Nucleic Acids Res. 38, D237–D243. 10.1093/nar/gkp970 19934256 PMC2808967

[B65] Price M. J. Carson A. R. Murray S. S. Phillips T. Janel L. Tisch R. (2012). First Pharmacogenomic Analysis Using Whole Exome Sequencing to Identify Novel Genetic Determinants of Clopidogrel Response Variability: Results of the Genotype Information and Functional Testing (GIFT) Exome Study. J. Am. Coll. Cardiol. 59, E9. 10.1016/S0735-1097(12)60010-2 22281261

[B66] Rasmussen-Torvik L. J. Almoguera B. Doheny K. F. Freimuth R. R. Gordon A. S. Hakonarson H. (2017). Concordance between Research Sequencing and Clinical Pharmacogenetic Genotyping in the eMERGE-PGx Study. J. Mol. Diagn. 19, 561–566. 10.1016/j.jmoldx.2017.04.002 28502727 PMC5500823

[B67] Reisberg S. Krebs K. Lepamets M. Kals M. Mägi R. Metsalu K. (2019). Translating Genotype Data of 44,000 Biobank Participants into Clinical Pharmacogenetic Recommendations: Challenges and Solutions. Genet. Med. 21, 1345–1354. 10.1038/s41436-018-0337-5 30327539 PMC6752278

[B68] Relling M. V. Klein T. E. (2011). CPIC: Clinical Pharmacogenetics Implementation Consortium of the Pharmacogenomics Research Network. Clin. Pharmacol. Ther. 89, 464–467. 10.1038/clpt.2010.279 21270786 PMC3098762

[B69] Sabater A. Ciudad C. Cendros M. Dobrokhotov D. Sabater-Tobella J. (2019). G-Nomic: a New Pharmacogenetics Interpretation Software. Pgpm Vol. 12, 75–85. 10.2147/PGPM.S203585 PMC6554524 31239753

[B70] Saminathan R. Bai J. Sadrolodabaee L. Karthik G. M. Singh O. Subramaniyan K. (2010). VKORC1 Pharmacogenetics and Pharmacoproteomics in Patients on Warfarin Anticoagulant Therapy: Transthyretin Precursor as a Potential Biomarker. PLoS One 5, e15064. 10.1371/journal.pone.0015064 21179214 PMC3001467

[B71] Sangkuhl K. Whirl‐Carrillo M. Whaley R. M. Woon M. Lavertu A. Altman R. B. (2020). Pharmacogenomics Clinical Annotation Tool (Pharm CAT ). Clin. Pharmacol. Ther. 107, 203–210. 10.1002/cpt.1568 31306493 PMC6977333

[B72] Santos M. Niemi M. Hiratsuka M. Kumondai M. Ingelman-Sundberg M. Lauschke V. M. (2018). Novel Copy-Number Variations in Pharmacogenes Contribute to Interindividual Differences in Drug Pharmacokinetics. Genet. Med. 20, 622–629. 10.1038/gim.2017.156 29261188

[B73] Schärfe C. P. I. Tremmel R. Schwab M. Kohlbacher O. Marks D. S. (2017). Genetic Variation in Human Drug-Related Genes. Genome Med. 9, 117. 10.1186/s13073-017-0502-5 29273096 PMC5740940

[B74] Schwarz U. I. Gulilat M. Kim R. B. (2019). The Role of Next-Generation Sequencing in Pharmacogenetics and Pharmacogenomics. Cold Spring Harb Perspect. Med. 9, a033027. 10.1101/cshperspect.a033027 29844222 PMC6360866

[B75] Shrestha S. Zhang C. Jerde C. R. Nie Q. Li H. Offer S. M. (2018). Gene-Specific Variant Classifier (DPYD-Varifier) to Identify Deleterious Alleles of Dihydropyrimidine Dehydrogenase. Clin. Pharmacol. Ther. 104, 709–718. 10.1002/cpt.1020 29327356 PMC6043412

[B76] Shu Y. Leabman M. K. Feng B. Mangravite L. M. Huang C. C. Stryke D. (2003). Evolutionary Conservation Predicts Function of Variants of the Human Organic Cation Transporter, OCT1. Proc. Natl. Acad. Sci. 100, 5902–5907. 10.1073/pnas.0730858100 12719534 PMC156299

[B77] Sivadas A. Salleh M. Z. Teh L. K. Scaria V. (2017). Genetic Epidemiology of Pharmacogenetic Variants in South East Asian Malays Using Whole-Genome Sequences. Pharmacogenomics J. 17, 461–470. 10.1038/tpj.2016.39 27241059

[B78] Suarez-Kurtz G. Parra E. J. (2018). “Population Diversity in Pharmacogenetics: a Latin American Perspective,” in Advances in Pharmacology (Elsevier), 133–154. 10.1016/bs.apha.2018.02.001 29801573

[B79] Svidnicki M. C. C. M. Zanetta G. K. Congrains-Castillo A. Costa F. F. Saad S. T. O. (2020). Targeted Next-Generation Sequencing Identified Novel Mutations Associated with Hereditary Anemias in Brazil. Ann. Hematol. 99, 955–962. 10.1007/s00277-020-03986-8 32266426 PMC7241966

[B80] Swen J. J. Nijenhuis M. de Boer A. Grandia L. Maitland-van der Zee A. H. Mulder H. (2011). Pharmacogenetics: From Bench to Byte- an Update of Guidelines. Clin. Pharmacol. Ther. 89, 662–673. 10.1038/clpt.2011.34 21412232

[B81] Tafazoli A. Wawrusiewicz-Kurylonek N. Posmyk R. Miltyk W. (2021). Pharmacogenomics, How to Deal with Different Types of Variants in Next Generation Sequencing Data in the Personalized Medicine Area. Jcm 10, 34. 10.3390/jcm10010034 PMC7796098 33374421

[B82] Taylor C. Crosby I. Yip V. Maguire P. Pirmohamed M. Turner R. M. (2020). A Review of the Important Role of CYP2D6 in Pharmacogenomics. Genes 11, 1295. 10.3390/genes11111295 PMC7692531 33143137

[B83] Thermofisher.Com/Pharmacoscan (2018). Verification of Buccal Swab and Saliva Sample Types for PharmacoScan Solution.

[B84] Twesigomwe D. Wright G. E. Drögemöller B. I. Da Rocha J. Lombard Z. Hazelhurst S. (2020). A Systematic Comparison of Pharmacogene star Allele Calling Bioinformatics Algorithms: a Focus on CYP2D6 Genotyping. NPJ Genomic Med. 5, 1–11. 10.1038/s41525-020-0135-2 PMC7398905 32789024

[B85] Twist G. P. Gaedigk A. Miller N. A. Farrow E. G. Willig L. K. Dinwiddie D. L. (2016). Constellation: a Tool for Rapid, Automated Phenotype Assignment of a Highly Polymorphic Pharmacogene, CYP2D6, from Whole-Genome Sequences. Npj Genomic Med. 1, 1–10. 10.1038/npjgenmed.2015.7 PMC5685293 29263805

[B86] Twist G. P. Gaedigk A. Miller N. A. Farrow E. G. Willig L. K. Dinwiddie D. L. (2017). Erratum: Constellation: a Tool for Rapid, Automated Phenotype Assignment of a Highly Polymorphic Pharmacogene, CYP2D6, from Whole-Genome Sequences. Npj Genomic Med. 2, 16039. 10.1038/npjgenmed.2016.39 PMC5685321 29266105

[B87] van der Lee M. Allard W. G. Bollen S. Santen G. W. E. Ruivenkamp C. A. L. Hoffer M. J. V. (2020a). Repurposing of Diagnostic Whole Exome Sequencing Data of 1,583 Individuals for Clinical Pharmacogenetics. Clin. Pharmacol. Ther. 107, 617–627. 10.1002/cpt.1665 31594036 PMC7027978

[B88] Van Der Lee M. Kriek M. Guchelaar H.-J. Swen J. J. (2020b). Technologies for Pharmacogenomics: A Review. Genes 11, 1456. 10.3390/genes11121456 PMC7761897 33291630

[B89] Walczak M. Skrzypczak-Zielinska M. Plucinska M. Zakerska-Banaszak O. Marszalek D. Lykowska-Szuber L. (2019). Long-range PCR Libraries and Next-Generation Sequencing for Pharmacogenetic Studies of Patients Treated with Anti-TNF Drugs. Pharmacogenomics J. 19, 358–367. 10.1038/s41397-018-0058-9 30293984

[B90] Wennerholm A. Dandara C. Sayi J. Svensson J. O. Abdi Y. A. Ingelman‐Sundberg M. (2002). The African-specific CYP2D6*17 Allele Encodes an Enzyme with Changed Substrate Specificity. Clin. Pharmacol. Ther. 71, 77–88. 10.1067/mcp.2002.120239 11823760

[B91] Westbrook M. J. Wright M. F. Van Driest S. L. Mcgregor T. L. Denny J. C. Zuvich R. L. (2013). Mapping the Incidentalome: Estimating Incidental Findings Generated through Clinical Pharmacogenomics Testing. Genet. Med. 15, 325–331. 10.1038/gim.2012.147 23196672 PMC3648626

[B92] Wouden C. H. Van Rhenen M. H. Jama W. O. M. Ingelman‐Sundberg M. Lauschke V. M. Konta L. (2019). Development of the PG x‐Passport: A Panel of Actionable Germline Genetic Variants for Pre‐Emptive Pharmacogenetic Testing. Clin. Pharmacol. Ther. 106, 866–873. 10.1002/cpt.1489 31038729 PMC6771671

[B93] Xie C. Yeo Z. X. Wong M. Piper J. Long T. Kirkness E. F. (2017). Fast and Accurate HLA Typing from Short-Read Next-Generation Sequence Data with xHLA. Proc. Natl. Acad. Sci. USA 114, 8059–8064. 10.1073/pnas.1707945114 28674023 PMC5544337

[B94] Yang Y. Botton M. R. Scott E. R. Scott S. A. (2017). Sequencing theCYP2D6gene: from Variant Allele Discovery to Clinical Pharmacogenetic Testing. Pharmacogenomics 18, 673–685. 10.2217/pgs-2017-0033 28470112 PMC5591463

[B95] Zhou Y. Fujikura K. Mkrtchian S. Lauschke V. M. (2018). Computational Methods for the Pharmacogenetic Interpretation of Next Generation Sequencing Data. Front. Pharmacol. 9, 1437. 10.3389/fphar.2018.01437 30564131 PMC6288784

[B96] Zhou Y. Mkrtchian S. Kumondai M. Hiratsuka M. Lauschke V. M. (2019). An Optimized Prediction Framework to Assess the Functional Impact of Pharmacogenetic Variants. Pharmacogenomics J. 19, 115–126. 10.1038/s41397-018-0044-2 30206299 PMC6462826

[B97] Zidan A. M. Saad E. A. Ibrahim N. E. Mahmoud A. Hashem M. H. Hemeida A. A. (2020). PHARMIP: An Insilico Method to Predict Genetics that Underpin Adverse Drug Reactions. MethodsX 7, 100775. 10.1016/j.mex.2019.100775 32123669 PMC7036477

